# Therapeutically applied Minecraft groups with neurodivergent youth

**DOI:** 10.12688/f1000research.129090.2

**Published:** 2023-11-20

**Authors:** Elizabeth Kilmer, Johnny Spangler, Jared Kilmer

**Affiliations:** 1 Take This, Seattle, WA, USA; 2 Game to Grow, Seattle, Washington, 98125, USA; 3 Antioch University, Seattle, WA, 98121, USA

**Keywords:** Minecraft, digital games, therapeutically applied minecraft, autism, ADHD, applied games, social skills

## Abstract

**Background:**

Therapeutically applied Minecraft groups are an intervention designed to support social engagement, confidence, and competence in youth. The flexible interaction format and use of a popular digital game support the fit of this intervention for use with neurodivergent youth. Minecraft is leveraged to support opportunities to build authentic relationships and social confidence in an engaging, low-stakes environment with peers. The group format allows for real-world social practice with peers, while the game environment can support motivation to interact with others, and provides multiple modes for such interaction (i.e., chat, building/movement with the avatar).

**Methods:**

This article outlines the theoretical foundations of therapeutically applied Minecraft groups as well as practical considerations for implementation. The method outlined includes the justification for this method, process of creating support groups, check-in and check-out processes, and in-game activity examples for different situations.

**Use Cases:**

Use cases are included to illustrate how the methods have been used in the past to support social growth with neurodivergent youth. Use cases include examples of different Minecraft servers, such as the habitat, and identifying stresses of social growth such as school anxiety and how the use of therapeutically applied Minecraft helped.

**Conclusions:**

Therapeutically applied Minecraft groups can provide opportunities for peer connection and social practice in a facilitated environment. Though the use of Minecraft and other games to support learning and social connection is prevalent in the media, the academic research in this area is sparse. This article provides an example of Minecraft in use in a therapeutic context and calls for more formal research in this area of therapeutically applied games.

## Introduction

Therapeutically applied Minecraft groups are an intervention designed to support social engagement, confidence, and competence youth and are particularly well-suited for use with neurodivergent youth, such as those with diagnoses of autism and/or ADHD. This group-based intervention utilizes the popular digital game Minecraft to create opportunities for building social capacity and confidence in an engaging, low-stakes environment with peers. The group format allows for real-world social practice with peers. The use of the game environment can support motivation to interact with others, especially for participants who are fearful or disinterested in peer interaction. Finally, the use of a digital game provides multiple modes for such interaction (i.e., chat, building/movement with the avatar), which creates greater autonomy in communication for the participants than a traditional in-person or virtual social skills group.

The reciprocal relationship between social connection and physical and mental wellness is well-documented ( Orben *et al.,* 2020; Viner *et al.,* 2012). Neurodivergent individuals, such as autistic individuals and those with attention-deficit/hyperactivity disorder (ADHD) are more likely than their neurotypical peers to experience peer rejection, loneliness, and social isolation ( Heiman *et al.,* 2015; Shea and Wiener, 2003; Unnever and Cornell, 2003; Orsmond *et al.,* 2013). In both ADHD and autistic individuals, loneliness has been found to increase the likelihood of depressive symptoms ( Hedley *et al.,* 2018; Houghton *et al.,* 2020). Challenges with social isolation became even more pronounced during the COVID-19 pandemic, though peer support was found to be a protective factor ( Laslo-Roth *et al.,* 2022; Sibley *et al.,* 2021; Pellicano *et al.,* 2022).

Challenges with communication and relationship building, especially in relation to neurotypical norms are intrinsic to the diagnostic criteria of both ADHD and autism spectrum disorder ( APA, 2022). Notably, though diagnostic and treatment norms have historically been focused on aligning individuals with neurotypical standards for social communication, newer interactionist/ecological neurodiversity approaches emphasize a shift in societal and environmental norms, while also supporting the development of skills adaptive to an individual’s current environment ( Dwyer, 2022). Consistent with these approaches and as challenges with peer communication can contribute to peer rejection and isolation, supporting neurodivergent adolescents’ social competence and confidence may be a valuable tool to reduce isolation and increase peer connection. Therapeutically applied Minecraft can provide opportunities for peer connection and social practice in a facilitated environment. Though the use of Minecraft and other games to support learning and social connection is prevalent in the media, the academic research in this area is sparse. This article is intended to outline the rationale for therapeutically applied Minecraft groups, share current use cases of the intervention, and highlight the need for further research in this area.

Minecraft is a first-person, sandbox-style, video game first developed by Mojang Studios in 2011 and purchased by Microsoft in 2014. Since its launch, Minecraft has expanded into a growing franchise that has enjoyed world-wide success, currently holding the title for best-selling videogame of all time ( Microsoft, 2021). Indeed, over 50% of youth (ages 9-11) in North America and Europe play Minecraft ( Microsoft, 2021). This widespread popularity makes interventions utilizing Minecraft especially well-suited for building social capacity that leverages shared interests and hobbies. In Minecraft, players have a customizable avatar they use to explore and interact with the procedurally generated world around them. Different modes (i.e., Survival and Creative) within the game allow for a customized user experience, providing players agency to adjust the amount of combat, challenge level to obtain materials, and ability to shape the world to fit their preferred playstyle. When used as a therapeutic intervention, the facilitator is empowered to adjust the environment and rules of the game to support player engagement and growth.

At its core, Minecraft is a building and exploration game, with mechanics that encourage creativity and nonlinear engagement. Minecraft has two primary styles of play, known as game modes, each emphasizing different aspects of player interaction within the virtual world. In Survival mode, players must find and gather resources in order to manage the health and hunger needs of their avatar, build structures, defend against hostile creatures, and navigate various hazardous locations. The challenges inherent in Survival are focused on player progression and resource gathering. For example, a new player must first make a wood pickaxe in order to gather stone, which in turn allows them to create a stone pickaxe, allowing them to mine iron. These resources must be found through exploration within the game world. In contrast, Creative mode resembles a complex digital LEGO set. Players have unlimited access to any resources in the game, they can fly around the world, place and break blocks easily, and the health and hunger mechanics are turned off. Creative mode incentivises building and creativity. Players can create fully interactive constructs without committing time to resource gathering including houses, spaceships, pixel art, or even recreate Middle Earth from *The Lord of the Rings* ( https://www.mcmiddleearth.com/). While each game mode highlights particular styles of play, there is no clear restriction between play style and game mode. Many popular Minecraft players on YouTube have become famous for expressing their creativity and building skill while playing in Survival mode (i.e., Technoblade, Dream, Mumbo Jumbo).

### The need for neurodivergent affirming social groups

With the appropriate support from qualified facilitators, Minecraft may be particularly well-suited to support the social capacity of neurodivergent youth. Neurodivergent individuals, such as those with ADHD or who are autistic, often face significant social stigma and rejection that can be detrimental to their mental health and well-being ( Bauminger *et al.,* 2003; Hedley *et al.,* 2018; Mueller *et al.,* 2012). Challenges with social communication and relationships, often associated with and exacerbated by the stigma of deviating from neurotypical standards of communication, can lead to feelings of rejection and isolation for autistic individuals ( Milton, 2012). Additionally, autistic and ADHD individuals are more likely to experience bullying and peer rejection than their neurotypical peers ( Paulson *et al.,* 2005; Maïano *et al.,* 2016).

In order to avoid social rejection and fit in among their neurotypical peers, many neurodivergent individuals intentionally camouflage or ‘mask’ their symptoms and behaviours (such as stimming, echolalia, and interrupting behaviour) to appear more neurotypical or “normative” ( Cook *et al.,* 2018; Dean *et al.,* 2017; Hull *et al.,* 2021; Pearson and Rose, 2021). Further, many traditional social skills training programs reinforce such masking through the notion that to be successful in community, educational, and employment settings individuals must ‘fit in’ and display neurotypical communication behaviours such as direct eye contact while speaking with others. However, despite the theoretical underpinnings of traditional social skills training programs, many autistic individuals have reported negative mental health outcomes related to this type of program and messaging, such as feeling disconnected from their sense of identity ( Shkedy *et al.,* 2020; Miller *et al.,* 2021). Additionally, though traditional social skill training programs have been widely popular, there have been recent criticisms. One criticism is that these programs can increase the knowledge of ‘social skills’ for neurodivergent youth, but they don’t self-reported or teacher-observed use of such skills ( Gates *et al.,* 2017).

As neurodivergent youth benefit from increased social networks and social support, identifying ways to support their social growth in a way that is affirming, engaging, and leads to real social connections is vital. Utilizing activities such as digital games that youth already find enjoyable and offer opportunities for social connection can create an excellent platform from which to scaffold social capacity. In this context, scaffolding refers to facilitator support through in-game environmental changes, intentionally designed activities, or interpersonal facilitation between platers in the group, participants may be able to engage in more challenging or complex social encounters than they would be able to do on their own. Some research has found that Minecraft may be more aligned with some neurodivergent individuals' natural communication styles as it allows for modes of communication aside from face-to-face interaction ( Ringland *et al.,* 2016). Additionally, with the continued prevalence of Minecraft as a hobby for youth, coupled with many adolescents’ active participation within at least one community server providing opportunities for social play, interventions using Minecraft can leverage pre-existing enthusiasm and social resources to enhance intervention outcomes. As socialization and connection between peers becomes commonplace in virtual settings, therapeutically applied Minecraft groups can create a naturalistic social setting to practice immediately relevant and meaningful social skills. Some of the aforementioned criticisms of traditional social skills groups can be addressed by shifting the context in which social skills practice occurs to a more real-world setting ( Gates e *t al.,* 2017). Further, shared backgrounds and interests can be a catalyst for motivating inter-group relationships and pro-social behaviour towards peers ( Daniel and Billingsley 2010; Grandin, 2019). For example, participants are able to build on a shared knowledge set (i.e., their interest and expertise in the game) while building rapport amongst each other.

Additionally, in the authors’ anecdotal experience, many neurodivergent adolescents report developing avoidance behaviours associated with social interactions, often stemming from multiple prior negative experiences with peers. Substantiating this, it has been proposed that social confusion and peer rejection related to autism presentation may increase social anxiety and social avoidance in autistic individuals ( Wood & Gadow, 2010), and qualitative research with male autistic adolescents support that previous rejection and fear of future rejection can lead to avoidance behaviors ( Acker *et al.*, 2018). Perceived asociality in neurodivergent individuals, especially autistic individuals, may be more representative of learned behaviour than an inherent lack of desire to connect with others. Interventions that set up peer networks for autistic students have been found to increase social engagement among their participants ( Hochman *et al*., 2015). However, simply creating a facilitated social space that respects individual communication needs may not inherently increase an individual's willingness to communicate with others, especially for individuals with high levels of social anxiety ( Acker *et al.*, 2018). Using a valued activity (e.g., Minecraft) can create both an added layer of safety stemming from the familiar environment, as well as an increased willingness to communicate with others in order to complete shared goals such as building a fortress or defeating a dragon.

Further, participation in social activities through a virtual environment creates opportunities to try new skills with potentially lower consequences for failure than a real-life setting, which may be particularly helpful for neurodivergent participants with a history of peer-rejection.

### Justification for Minecraft as a platform for therapeutic intervention

Although a number of video games and other platforms can provide a virtual space for individuals to interact, the authors of this article posit that Minecraft is a particularly auspicious choice for therapeutic application due to several key components. Minecraft has precedent of use in educational and therapeutic contexts, is a popular multiplayer game with multiple game modes, and contains seemingly limitless opportunities for customizing the in-game experience.

The first-person, sandbox-style of Minecraft has been utilized in educational and therapeutic contexts alike. Interventions using Minecraft have previously been designed to support cooperative communication among autistic youth ( MacCormack and Freeman, 2019). MacCormick and Freeman’s ‘virtual environment social program’ is a heavily structured intervention that utilizes the framework of Legoff’s LEGO therapy in the Minecraft virtual setting to support strategic communication with autistic youth ( LeGoff, 2004; LeGoff *et al.*, 2012). Similar to LEGO therapy, participants take turns in specific roles (i.e., architect, artist, and foreman) to create buildings ( MacCormack and Freeman, 2019). Together participants choose a structure to build in the game, and then work together within their predefined roles to make the building. In addition to autism specific groups, Minecraft has been utilized in community building programs, such as the ‘Minecraft Gaming Day’ developed by a library in Melbourne ( Cilauro, 2015). Minecraft has been further utilized to support the development of conflict-resolution skills ( Jagannath *et al.*, 2020), social learning, and emotional regulation skills in adolecents ( Zolyomi & Schmalz, 2017). Minecraft’s ‘Education Edition’ has supported the expansion of Minecraft to support social-emotional and academic curricula in schools ( Baek *et al.,* 2020; Cheng, 2016). In addition to group-based interventions, Minecraft has also been utilized in the context of individual therapy ( Finch, 2021).

Perhaps a key component in supporting such educational and therapeutic uses, Minecraft is popular. In North America and Europe, over 50 percent of kids ages 9-11 play Minecraft and it has over 140 million active monthly users ( Microsoft, 2021). When identifying a game to utilize within a therapy setting, using a game that participants are likely to be familiar with, can increase participant motivation and willingness to engage, as well as create opportunities for the participant to be an expert in the area. Minecraft may be an especially good fit when working with neurodivergent individuals, as there are multiple Minecraft servers and communities specifically designed for neurodivergent individuals such as Autcraft ( https://www.autcraft.com/), designed for youth with Autism.

In addition to its current popularity, Minecraft has demonstrated longevity within the zeitgeist - the game has been released for over a decade and continues to maintain popularity within adolescent demographics. With the learning curve and effort involved in implementing game-based therapeutic interventions, the game used should have some expectation of longevity. Additionally, the prevalence of Minecraft in the culture creates a natural bridge between skills learned within the therapeutic setting and other experiences. One example of this generalization can be seen in an anecdote one of the authors using vocabulary from Minecraft to connect with their student. A student was having a difficult time expressing their emotions as they were getting more frustrated in a social situation. The teacher asked, “are you feeling Creeper level frustrated, or are you more of a Zombie right now?” The student thought about it and answered “Actually, I am Angry Pigman angry; I am ok but if someone touches me I am going to attack them.” The cultural ubiquitousness of Minecraft knowledge not only gave this student a shared language to express how they were feeling, but it also encouraged them to reflect on the *type* of frustration they were feeling and express how it presented externally.

Minecraft has multiple game modes, a multitude of community-designed customization options, and no clearly defined narrative or in-game goals. As such, it can be used as an open-world play-space for therapeutic applications, similar to a well-stocked play therapy room or the stand tray in sand tray therapy. The facilitator can customize the world to create guides and boundaries as appropriate to support participation in group activities. For example, the group could start in a standard generated world and work together to build a secret hideout. For groups that benefit from a more visual building prompt or guidelines, the facilitator could build a series of floating islands over an ocean and invite each player to customize their own island. The second option creates visual boundaries that may support players staying in the same area on the map, increasing opportunities for collaboration.

In contrast to a physical play space, facilitators have increased control over the environment of the Minecraft game space. For example, if a participant becomes frustrated and uses their avatar to damage the in-game constructions of another player, the participant or facilitator can typically fix the damage to the construction with minimal effort. In contrast, the repair of such damage may not be possible to the same extent in the real world due to limited resources (i.e., time, money, material). The use of games or simulation to practice skills in contained environments is common practice in education and medical fields, and skills practiced in such simulated environments, especially when the simulation is responsive to player input, have been found to translate to real-world use and competency ( Sturm *et al.,* 2008; Ward *et al*., 2006).

As of this writing, Minecraft has three editions - Java, Bedrock, and Education. Java Edition is the most customizable of the three, with immense community support through mods, plug-ins and tutorials. Java is only available on PC and requires a significant amount of computer processing power. While Java offers greater access to group management and player safety features (such as being able to undo player actions), this edition requires the use of mods and plug-ins to optimally function. This results in a higher set-up time cost and level of technical knowledge by the facilitator. In contrast, Education Edition was designed for use in schools, and designed to be implemented by educators with little prior knowledge of Minecraft for use in classrooms and after school clubs. Minecraft Education Edition has premade activities and more safety features innately enabled, but fewer opportunities for customization by the facilitator and players. Though Education Edition can be an excellent option for many facilitators newer to Minecraft or with less time to invest in learning technical skills, some experienced players may be frustrated by the lack of customizability. Finally, Bedrock Edition is designed to be playable across a multitude of devices, including PC, gaming systems, mobile devices, and even VR. The drawback to Bedrock is that it has the least amount of customizability of the three. The therapeutically applied Minecraft groups outlined in this article utilize the Java edition.

## Methods

Therapeutically applied Minecraft groups can be delivered virtually or in-person. When delivered virtually, participants should be in a private, shared voice and video chat to supplement the in-game opportunities for communication (e.g., chat). Among in-person groups, the ideal setup allows participants to see and hear each other with minimal effort - for example, participants can play on laptops around a shared table. Each session, group members meet to collaborate on shared goals that require communication, regulation, and planning skills. Goals for each group will vary based on the needs and preferences of the members, and generally fall under the umbrella of social confidence and social capacity building. Groups consist of three to five participants and one facilitator. Participants typically meet for 90-minute weekly sessions for nine-12 weeks. Re-enrolment in groups can be appropriate when it supports participant goals, such as to deepen and maintain friendships. The structure of every group includes a check-in (10 minutes), in-game activities (75 minutes), and a check-out (five minutes).

Facilitators of therapeutically applied Minecraft groups should have training and experience with the needs and experiences of the group members, facilitating group-based interventions, and managing a Minecraft server. Though specific degrees or licensure are not required in many areas for therapeutic (as opposed to psychotherapy) services, facilitators should seek appropriate training and/or supervision if operating on the edge of or outside their area of competency. Specific requirements for training and education of facilitators delivering therapeutic or psychotherapy services vary by state, and facilitators should adhere to any requirements in the area in which they practice. The facilitator of the therapeutic Minecraft groups explored in the use cases in this article is a licensed mental health counselor in the state of Washington, with extensive experience working with neurodivergent youth in group-based settings.

Each participant must have their own device that runs Minecraft in order to participate in the group activities. Whether participants are meeting in person or virtually, they play together by connecting to a Minecraft server which runs the shared world they will be playing in. Servers are a separate computer that players connect to through a local network or over the internet. Though it is possible to join someone else's personal world without the use of a server, which is often how Minecraft is used in one-on-one therapeutic settings, when running a group session it is necessary to have a central computer that hosts a shared world. This gives the facilitator more control over the experience of the participants and is the most stable option when it comes to connecting multiple participants in a single Minecraft world.

### Check-in

**Figure 1.**
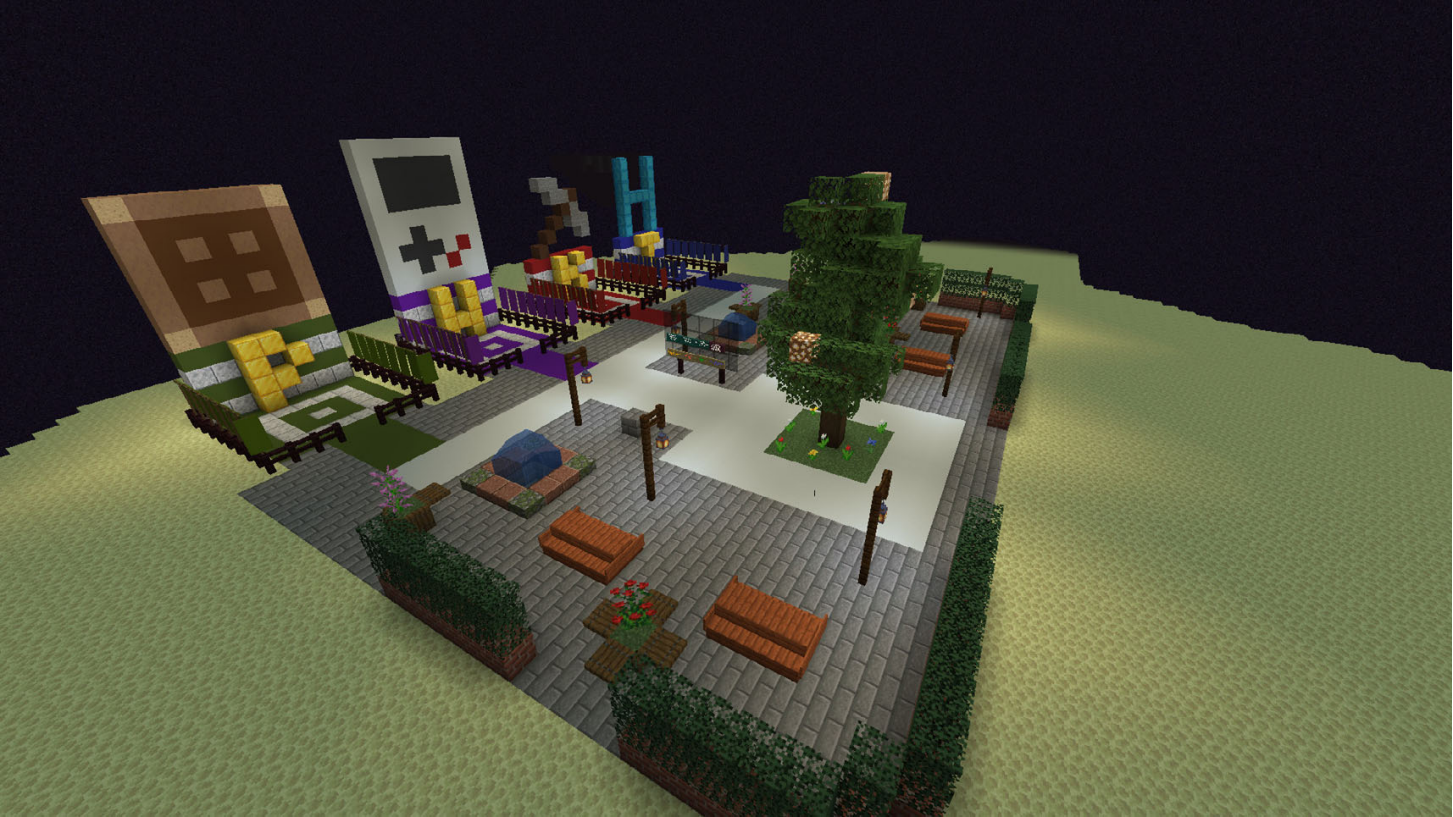
Example of a Minecraft waiting room.

During the group check-in, participants answer a check-in or warm-up question to support their orientation to the group and create an opportunity to learn more about the other participants through a structured prompt. Though the question changes each session, the consistent routine of the check-in can create structure that supports the ambiguity of the changing question. This routine can be particularly beneficial to neurodivergent individuals as the changing check-in question is embedded in a known routine. The check-in process typically takes approximately five minutes and marks the formal start of the group. While participants are answering the check-in question, their game avatars can be in the Minecraft server, and should be in a minimally engaging environment. Participants may be eager to join the game and start playing, so having a consistent ‘waiting room’ space inside the in-game environment allows participants to be in the game as the group starts (e.g., a small floating island in outer space). An example of a waiting room can be seen in Figure 1. The consistent waiting room space can create an engaging enough environment for participants to stay in the space during check-in but should not be so engaging as to distract from the check-in process. When working with neurodivergent adolescents in-particular, the authors have found that ‘blank room’ waiting spaces can lead to participants leaving the shared server and logging into a personal Minecraft server, however the ‘waiting room’ often has enough visual stimuli that when coupled with the predictable routine of check-in to in-game activities can support player engagement.

### In-game activity examples

Following the check-in, the group moves to the in-game activities. Depending on the interests and needs of the group, participants may engage in one activity for an entire session, or switch between two activities. Additionally, it is common for groups to have ongoing goals they can return to across sessions. These goals often are related to larger building or strategy projects. There are a multitude of in-game activities that can be used to support group cohesion and player growth, with varying levels of preparation and facilitator technical skill required. The authors have highlighted three commonly used activities in therapeutically applied Minecraft groups.

*Maze swap - creative mode activity*

Creative mode allows for low-stakes opportunities for collaboration and creativity, without the stress of resource management or time pressures. An activity that uses the Creative game mode is ‘Maze Swap’. In this activity, the group is broken into two teams. Each team is tasked with building a maze that another team will attempt to complete. They are given a time limit and the prompt that the maze needs to be ‘fun, challenging, and engaging’. After the building time has elapsed, the teams swap locations and attempt to complete the other team’s maze. This activity focuses primarily on the skills of group collaboration, communication, and perspective taking. While each participant can take their own section of the maze to work on or work together on each section, they still need to coordinate with their peers to make sure that areas connect and that there is an overarching structure to the build, with a clear beginning and end. The structure of this activity allows participants to choose their level of peer interaction. This allows players to fully participate in the activity regardless of their level of social comfort and increase their interaction with peers as they become more interested and comfortable. Within this activity, the participants are encouraged to be creative and add their own style to the build, giving them a sense of ownership with the final product. The autonomy participants’ have in the design and building process can support participant investment in the activity and feedback process. Throughout the activity, facilitators can prompt questions and conversations about the intent and expected impact of design choices, creating opportunities for participants to engage in perspective taking about the other team’s experience. Participants are encouraged to put themselves in the shoes of the players who will be going through their maze. They are asked questions like ‘Is any part too hard or frustrating?’ or ‘How clear are the goals of the maze?’. After the teams go through each other’s mazes, they are brought back together to debrief the experiences and offer feedback on what was ‘fun, challenging, and engaging’, giving the teams the opportunity to understand how well each team’s intentions for their mazes aligned with the actual play experience.

*Laser tag - minigame example*

Minigames are games created within Minecraft, using its mechanics to facilitate player experiences in a highly targeted way. These activities are typically set up in the game ahead of time by the facilitator, and players are transported to a specific area within which to participate in the game. Minigame activities are commonly found on public Minecraft servers, and these activities can be adapted within private servers for therapeutic use. One example of a minigame is laser tag. In this game, participants are divided into two teams, given a bow, and placed in an enclosed arena with the goal of hitting the other team with their arrows. Once hit, players are returned to ‘spawn’ where they can re-enter the arena and continue playing. An in-game scoreboard keeps track of hits, and each team tries to earn the most points. Similar to team sports, this activity can support frustration tolerance, planning, and strategic communication skills. This activity functions well as an intermezzo between more focused activities or as a group rapport building activity. As laser tag and many other minigames are competitive, facilitators should be mindful of participant’s skill with minigames, their frustration tolerance, and response to failure. If participants find the game too challenging or overwhelming, participation in such an activity may damage rapport instead of supporting its growth.

*Quest board - survival example*

Survival mode is well-suited for activities that require planning, time management, and collaboration. Players have fewer abilities and limited resources than in creative mode, and survival mode requires ongoing character management of health and hunger. An example of a Survival mode activity is ‘Quest Board’. In this activity, participants are given a new world to explore. The basic mechanics of the game require them to start gathering materials and building shelters. Participants can gather resources and build their shelters independently or collaborate with other group members, which maintains options for participant autonomy. The facilitator creates a board in the game prior to the group start with specific quests and rewards. The quests are geared towards particular styles of engagement that are growing edges for the participants. Examples of tasks can be ‘find a flower that is the favourite colour of another player’ or ‘build a house that has a room for each player’. These tasks emphasize reciprocal communication and collaboration.

### Check-out

The check-out process allows for a consistent and predictable transition from the game space to the end of the group and the real world. This routine transition can be particularly helpful for neurodivergent youth, who may struggle to switch tasks. During the check-out process, the participants and facilitator answer three questions in the same order. Unlike the check-in question, the check-out questions do not vary from week to week. The three questions are ‘What is a spotlight you would like to shine on someone else? Something they did that enhanced your game today’, ‘What was a challenge or something you learned?’, and ‘What is something you’re looking forward to for the next group?’. These questions allow participants to reflect on their play and draw connections between their experience and the other players. The facilitator can use their own response to highlight participant actions, model self-reflection, and reframe challenging scenarios.

## Use cases

### Ethical statement

The following use cases share examples of participant experiences in Therapeutically applied Minecraft groups. All participant names have been changed to protect their privacy, and other identifying information has been omitted. Participants discussed in these use cases were asked for permission to share their stories and written informed consent to share the below stories was obtained from participants’ legal guardians. Data from these groups was not formally collected to be assessed for validated, but in some cases instances were noted to demonstrate the potential effectiveness of this method. The below use cases fall under Antioch University’s definition of ‘Individual Case Studies’, do not meet criteria as human subjects research, and did not require IRB review.

### ‘The habitat’

A group consisting of five participants, ages 10-12 met online for weekly group sessions. The participants identified as neurodivergent and also reported a history of difficulty with peer communication and rapport building. Communication was facilitated through video chat and group activities happened within a shared Minecraft world. Four of the participants had been meeting for several months, and a new participant was joining the group. The facilitator’s goals were to help the new participant orient to the group norms while encouraging the other participants to incorporate new perspectives into their activities. To support a successful integration of the new participant to the group and allow the participants to increase their communication at a rate comfortable to them, the facilitator selected an activity that began with parallel play, with opportunities for increased communication and collaboration as the players were ready. Players were each given an animal and were asked to create a ‘habitat in a box’ for the animal within a specified amount of time. The time limit, type of animal, and the scope of the habitat created clear guidelines for the players to then utilize their creativity to determine what the best ‘habitat’ was for their animal. While the participants were building, the facilitator encouraged the participants to talk through their build and creative thought process, keeping a focus on the goal of the activity while allowing participants to take cues from each other. While the experienced participants engaged in the activity without hesitation, Max ^1^, the new participant, was not immediately interested in the activity and began flying around the world. At one point Max began interacting with other habitats by filling them with animals, which upset the other participants. When it was pointed out to Max that his actions were making others upset, he was visibly upset and it was clear to the facilitator that he had not expected his actions to have this reaction from others. The facilitator used a perspective taking intervention, asking Max if he was aware the habitat box belonged to someone. Max responded “I guess so, but I didn’t think about it. It just looked so cool I wanted to use it, I’m really sorry I ruined it.” Max’s statement allowed the other group members to better understand Max’s intent and decision-making process. This, combined with the compliment about how ‘cool’ the box was, supported the repair and re-engagement process with the other group members. The facilitator then supported the players in reflecting on what it can feel like to be in a new group and not understand the expectations yet. Participants were asked if they ever made a mistake and hurt someone’s feelings, and how that felt. The facilitator removed all the dogs, restoring the build to its original form, and Max then flew off to make his own ‘dog box’. This is a particularly poignant example of a facilitated social engagement because Max had had a history of being kicked out of social groups due to ‘bullying behaviour’. The flexibility of Minecraft, paired with strong facilitation skills, allowed him to experience rupture and repair within a social setting, while also providing perspective taking opportunities for the other participants. While this experience could have happened in another social setting with a skilled facilitator, Minecraft offered both an easily accessible shared space for players to engage within, along with the lower stakes that comes from a virtual space that the facilitator has technical control over.

### School anxiety

In addition to supporting in-group communication, Minecraft can be used as a medium to create shared language accessible to younger participants. This group consisted of four participants who connected through video chat and a shared Minecraft world. In anticipation of the participants returning to school after summer vacation, the facilitator chose an activity designed to help the participants process their emotions around the coming change. The participants were given a pre-made model of a school within Minecraft. It consisted of hallways, lockers, classrooms, bathrooms, big front doors, and a principal's office. The participants were asked to pick a classroom and make it look like the one at their school. They were also given the option to work on the rest of the school once they were finished. Once the allotted time had finished, the facilitator took the group around on a tour of the classrooms while each student described their design process for each classroom. One classroom had been filled with Minecraft villagers, who wander around and make noise. Through subtle guidance and prompting from the facilitator, the participant was able to describe how their classroom was “always really noisy” and that they often felt overwhelmed in the space. Other participants chimed in with similar experiences and spontaneously started sharing ideas for how to help with the noise. The session ended with the group working together to cover the classroom with wool blocks (to reduce the noise) and corralling all the villagers out into the hallway so they could have the classroom to themselves. Through creating a visual representation of their anxiety, the participants were able to experience a commonality in shared struggle. During the debrief, the facilitator highlighted the skills that the participants used and helped generalize the experience into strategies they could use in an actual classroom (e.g., noise cancelling headphones, a quiet corner).

### Challenges of implementation

Common challenges when using Minecraft as a therapeutic tool can be generalized into the broad categories of practical, technical, and ethical. Practical challenges include such things as access to appropriate devices that can run the game, participant expectations, and maintaining a clear and consistent communication space (especially when meeting remotely). Technical challenges can include connecting participants to the shared world, the skill curve of in-game commands and controls, managing the built-in safety features and/or adding additional ones through modification of the game, and troubleshooting technical problems as they arise. Ethical challenges specific to using Minecraft are primarily focused around maintaining privacy within a game that is designed to facilitate social interaction and friendships. While the Education Edition is not socially oriented, it is unclear if appropriate levels of security, or a business associates agreement (BAA), are available from Microsoft, which would be important for clinical/medical settings. Across settings, facilitators should be properly trained to offer intentional interventions while maintaining participant safety with the given population.

### Potential risks of therapeutically applied Minecraft

Minecraft was not designed as a therapeutic intervention and requires a significant facilitator knowledge to correctly implement the safety tools necessary for their group. Such safety tools can include technical interventions such as turning off player damage or the ability to “undo” in-game player actions. Because activities take place in a virtual world, the biggest risk to players is within social/emotional experiences, such as being a target for bullying or feelings of being excluded or marginalized. The flexibility and ease of access that Minecraft offers as a tool also lends itself to abuse by participants if good boundaries are not maintained. Minecraft has mechanics such as TNT and explosive creatures, which can allow participants to quickly destroy the creations of their peers. Though there is no research currently available on the risks of therapeutically applied Minecraft groups, anecdotes suggest if a facilitator is unprepared to navigate the technical or social aspects of the group, they may fail to maintain a safe enough environment for the players, leading to unnecessary emotional distress of players.

### Recommendations for future research

Therapeutic applications of video games, particularly among games that were not designed to be therapeutic (e.g., Minecraft), is an emerging area of research. Though the rationale for the use of such games in conjunction with established therapeutic techniques is clear from a theoretical lens, more research is needed in this area to better understand the potential risks and benefits of such interventions.

Historically, research into youth experiences and interventions, especially among neurodivergent adolescents, has centred on the reports of parents, teachers, and other adult observers. To better understand the true impact of interventions on neurodivergent adolescents, the feedback of adolescents should be centred accordingly. A mixed-methods approach with opportunities for both qualitative and quantitative data should be used to understand the impact of novel interventions and direct future research. For example, tape review of sessions to examine changes in reciprocal communication between participants over time combined with, self- and observer-report measures with additional open-ended questions completed at regular intervals to track change over time. These measures should include information about real-world changes in behavior (or lack-thereof). Common goals for future research groups will vary by population and cohort, but measures selected should target the goals of the group.

## Conclusions

The use of valued hobbies to support participant growth, as well as the utilization of simulations to practice new or atrophied skills are not new concepts in mental health. Advances in technology and digital games can be utilized to support adolescent social capacity and connection. As social connection is important for well-being, and neurodivergent youth have higher rates of peer victimization and loneliness, identifying strategies specifically targeted to these populations is important.

Therapeutically applied Minecraft is an intervention designed to support the social growth of neurodivergent individuals with respect to participant communication preferences and individual autonomy. In therapeutically applied Minecraft groups participants meet weekly for 90 minutes to engage in facilitated activities in a virtual world. As outlined through the use cases in this article, the game environment allows for low-stakes social practice in a valued activity, which may support motivation and engagement in social activities. Furthermore, though Minecraft is particularly well suited to support neurodivergent youth social development, other multiplayer digital games with collaborative play styles can also be appropriate for such interventions and use many of the same techniques discussed in this article.

Though there is currently little academic research on the therapeutic applications, Minecraft has been increasingly popular in community and educational settings, and future research should focus on both the possible benefits as well as potential risks of such interventions with neurodivergent youth.

## Data Availability

All data underlying the results are available as part of the article and no additional source data are required.

## Footnotes

^1^
Names of participants have been changed to protect participant privacy as outlined in the ethical statement.
