# Supplementary material for: Therapeutically applied Minecraft groups with neurodivergent youth
Source: F1000Res. 2023 Nov 20;12:216. Originally published 2023 Feb 27. [Version 2] doi: 10.12688/f1000research.129090.2 (PMC10958151; doi:10.12688/f1000research.129090.2)
Supplement: Peer review history

# Reviewer report: Reviewer response for version 2

**DOI:** 10.5256/f1000research.159004.r247758

**Published:** 2024-03-21

**Authors:** Rayner Bin Tangkui

**Affiliations:** 1 Department of Planning, Research and Innovation, Institute of Teacher Education Keningau Campus, Keningau, Sabah, Malaysia

The article presents an innovative approach to supporting neurodivergent youth through therapeutically applied Minecraft groups. While the rationale and description of the method are compelling, the article would benefit from additional technical details for replication, a stronger empirical foundation, and formal research to support its conclusions. Addressing these areas will significantly enhance the article's scientific soundness and its value to practitioners and researchers interested in digital game-based interventions for neurodivergent populations.

Is the rationale for developing the new method (or application) clearly explained?

Yes

Is the description of the method technically sound?

Partly

Are the conclusions about the method and its performance adequately supported by the findings presented in the article?

Partly

If any results are presented, are all the source data underlying the results available to ensure full reproducibility?

No source data required

Are sufficient details provided to allow replication of the method development and its use by others?

Partly

Reviewer Expertise:

Digital-Game Based Learning, Minecraft-Based Learning,

I confirm that I have read this submission and believe that I have an appropriate level of expertise to confirm that it is of an acceptable scientific standard.

# Reviewer report: Reviewer response for version 1

**DOI:** 10.5256/f1000research.141749.r187088

**Published:** 2023-08-08

**Authors:** Siva Priya Santhanam

**Affiliations:** 1 Department of Communication Sciences and Disorders, College of Health and Human Services, Bowling Green State University, OH, Bowling Green State University, Bowling Green, Ohio, USA

This Method Article is useful as it adds to the existing literature on the value of videogames in supporting socialization among neurodivergent youth. The authors have described Minecraft as a tool to be used to support socialization among autistic youth. They described how the groups are conducted with a facilitator and provide a case example. They then summarize how the groups support socialization and reduce anxiety. Not much supporting evidence is provided in the discussion on anxiety reduction, but the focus is on how the Minecraft platform has the potential to reduce anxiety. They conclude the paper with future suggestions on how Minecraft can be utilized therapeutically.

 Please see comments below to help improve the manuscript.

 The format of background, method, and results does not quite fit this manuscript as it is an Method Article and not a Research Article based on empirical data. Align the abstract format with the format of the manuscript with the first paragraph starting with the purpose of the paper, a description of Minecraft, and its usefulness as a friendship intervention. Describe the method on how it is used in the method section. There are really no results for this paper. So, the abstract can be concluded with suggestions on how to implement the groups if that was the purpose of the article.

 It would be helpful to state the purpose clearly. Is the purpose to teach others how to facilitate the groups? Or to describe the value of Minecraft in therapeutic settings?

 Does your group focus on autistic youth only or do individuals of other neurodivergent identities join the groups as well?

 In this sentence, *“Therapeutically applied Minecraft groups are an intervention designed to support social engagement and growth in youth and are particularly well-suited for use with neurodivergent youth.”* Are the “groups” an intervention? Please define what the intervention entails within this group. Perhaps you may want to say, “Minecraft, a commercially available digital game, serves as an intervention tool that supports social engagement.” When you use the word “growth in youth”, please mention growth of what?

 What does scaffolding in Minecraft groups look like? Who does the scaffolding? Or who is ideally qualified to do the scaffolding? What training do they receive?

 Please use the copyright symbol © where needed – Minecraft, Microsoft etc.

 The discussion of “practicing social skills” is contradictory to the neurodiversity paradigm in that it describes autistic people as those who have “deficits” in communication.

 It would be helpful to elaborate on what really is the purpose of this article besides describing the use of Minecraft therapeutic intervention.

 It is not clear to me how the paragraph on masking is connected to the previous paragraph on page 4. A discussion and understand of masking and how it is associated with social isolation is helpful and necessary, but it has not been clearly laid out.

 Please provide a citation for this sentence “ *Many neurodivergent adolescents report developing avoidance behaviours associated with social interactions, often stemming from multiple prior negative experiences with peers.”*

 Please provide a citation for this sentence *“However, simply creating a facilitated social space* *that respects individual communication needs may not inherently increase an individual's willingness to communicate with others.”*

 In the paragraph on existing uses of Minecraft and the paragraph on justification for use of Minecraft, there are some overlapping points. Please consider combining the two paragraphs. It might also be helpful to explore how Minecraft is used in therapeutic settings outside of supporting autistic individuals. For example, these articles discuss the use of Minecraft in other scenarios. Using these to support the paragraphs would be a valuable addition. The article links are provided here. Mining for Social Skills: Minecraft in Home and Therapy for Neurodiverse Youth; Gliosci & Barros Pontes E Silva ^1^; Jagannath, Salen & Slovàk ^2^.

 Further in the paragraph on justification, there are points that also tie in with points in the previous paragraph on applications with neurodivergent individuals. So, I encourage the authors to re-read and consider rewriting these sections to be mutually exclusive paragraphs of the essay.

 The method of implementation is clear with case examples. Practical suggestions on how to facilitate the session would be helpful to add here for others to facilitate the groups if that was the intended purpose of the article.

 Suggestions on what facilitation entails would be helpful for someone to replicate the groups as well. What is the main goal of facilitation?

Is the rationale for developing the new method (or application) clearly explained?

Yes

Is the description of the method technically sound?

Partly

Are the conclusions about the method and its performance adequately supported by the findings presented in the article?

Partly

If any results are presented, are all the source data underlying the results available to ensure full reproducibility?

Partly

Are sufficient details provided to allow replication of the method development and its use by others?

No

Reviewer Expertise:

Supporting autistic adults' social communication through gaming.

I confirm that I have read this submission and believe that I have an appropriate level of expertise to confirm that it is of an acceptable scientific standard, however I have significant reservations, as outlined above.

## References

[1] : Therapeutic Interventions with Videogames in Treatments for Depression: A Systematic Review. *Games Health J* .2023;12(4) : 10.1089/g4h.2022.0094 269-279 10.1089/g4h.2022.0094 36946750

[2] : "(We) Can Talk It Out...": Designing for Promoting Conflict-Resolution Skills in Youth on a Moderated Minecraft Server. *Proceedings of the ACM on Human-Computer Interaction* .2020;4(CSCW1) : 10.1145/3392855 1-26 10.1145/3392855

# Response

**Published:** 2023-11-15

**Authors:** Elizabeth Kilmer

Authors’ response to reviewer with changes made for V2 of article. Reviewer commends in bold. 

**This Method Article is useful as it adds to the existing literature on the value of videogames in supporting socialization among neurodivergent youth. The authors have described Minecraft as a tool to be used to support socialization among autistic youth. They described how the groups are conducted with a facilitator and provide a case example. They then summarize how the groups support socialization and reduce anxiety. Not much supporting evidence is provided in the discussion on anxiety reduction, but the focus is on how the Minecraft platform has the potential to reduce anxiety. They conclude the paper with future suggestions on how Minecraft can be utilized therapeutically.**

**Please see comments below to help improve the manuscript.**

**The format of background, method, and results does not quite fit this manuscript as it is an Method Article and not a Research Article based on empirical data. Align the abstract format with the format of the manuscript with the first paragraph starting with the purpose of the paper, a description of Minecraft, and its usefulness as a friendship intervention. Describe the method on how it is used in the method section. There are really no results for this paper. So, the abstract can be concluded with suggestions on how to implement the groups if that was the purpose of the article.**

*We appreciate the reviewer’s concern that the standard abstract format outlined by F1000 guidelines doesn’t appropriately fit this manuscript. We’ve adjusted the abstract as recommended by the reviewer.*

**It would be helpful to state the purpose clearly. Is the purpose to teach others how to facilitate the groups? Or to describe the value of Minecraft in therapeutic settings?**

*The purpose of this article is to describe a current use of Minecraft in therapeutic settings and encourage increased research in this area. We’ve clarified this purpose in the abstract, introduction and conclusion section of the article.*

**Does your group focus on autistic youth only or do individuals of other neurodivergent identities join the groups as well?**

*This group is open to youth with goals of increasing social confidence and capacity, and no specific diagnosis is required. The majority of participants across these groups have a diagnosis of autism, ADHD, and/or an anxiety related disorder, and for this paper we’re focusing more specifically around autism/ADHD diagnoses. We’ve clarified this language throughout the paper.*

**In this sentence, *“Therapeutically applied Minecraft groups are an intervention designed to support social engagement and growth in youth and are particularly well-suited for use with neurodivergent youth.”* Are the “groups” an intervention? Please define what the intervention entails within this group. Perhaps you may want to say, “Minecraft, a commercially available digital game, serves as an intervention tool that supports social engagement.” When you use the word “growth in youth”, please mention growth of what?**

 The groups are the intervention, and they utilize the game Minecraft to do so – we’ve adjusted our language to better clarify our meaning here. We’ve removed the phrase “growth in youth” and replaced it with more specific language.

**What does scaffolding in Minecraft groups look like? Who does the scaffolding? Or who is ideally qualified to do the scaffolding? What training do they receive?**

*We’ve clarified language around scaffolding in therapeutically applied Minecraft groups and have added more information about recommendations for facilitator qualifications.*

**Please use the copyright symbol © where needed – Minecraft, Microsoft etc.**

*The manuscript is written in APA style, which does not use trademark symbols. We did make sure to identify the original studio as well as acknowledge its acquisition and ownership by Microsoft, which is done on page three.*

**The discussion of “practicing social skills” is contradictory to the neurodiversity paradigm in that it describes autistic people as those who have “deficits” in communication.**

*We understand that discussion around “practicing social skills” can be a contentious one in the neurodivergence community, especially given how the practice of social skills has often been applied in an oppressive manner to force autistic people to conform to allistic standards. We appreciate the concern and feedback from the reviewer and have worked to adjust our language to increase clarity. We would like to note that one of the authors is ADHD and another autistic; we have personal experience with the harm that oppressively applied interventions can cause by an over-emphasis on fitting in and the focus on a medical/deficit-based model of disability. We use the word "disability" in this case due to the wildly disparate - and sometimes contradictory - ways people apply the term "neurodiversity." Though we do not claim to speak for all disabled or neurodivergent individuals, our experiences both personally and professionally continually reinforce the value of teaching and practicing social skills in ways that respect individual autonomy and preference. We believe that most individuals, regardless of disability or neurodivergent status, can benefit from learning more about communication and practicing skills in a judgement-free environment which is supportive of their individual goals. We’ve added in additional language to clarify our alignment with an interactionist/ecological neurodiversity approach. Though beyond the scope of this paper, we believe that a failure to acknowledge challenges, needs, or impairments in communication is at best naively idealistic and at worst actively ableist. To put it simply, ignoring an individual’s needs and impairments can deny them access to accommodations.  Autistic and ADHD brains don’t need to be “fixed,” but we may need additional support or practice in some skills, including those that allow us to communicate with others more effectively (social skills), especially in how the other people can help accommodate our differing needs. The onus on communication should not be placed solely at the feet of neurodivergent individuals, but neither should we be denied skills in support of an ideal that aims to absolve us of disability.*

**It would be helpful to elaborate on what really is the purpose of this article besides describing the use of Minecraft therapeutic intervention.**

*The primary purpose of the article is to describe the use of Minecraft in therapeutic settings. As there is incredibly sparse information in the literature, the authors hope is that this article will help to illustrate a use of Minecraft in a therapeutic setting and inspire further research in the area.*

**It is not clear to me how the paragraph on masking is connected to the previous paragraph on page 4. A discussion and understand of masking and how it is associated with social isolation is helpful and necessary, but it has not been clearly laid out.**

*We’ve added language to further clarify how masking is associated with social isolation.*

**Please provide a citation for this sentence “ *Many neurodivergent adolescents report developing avoidance behaviours associated with social interactions, often stemming from multiple prior negative experiences with peers.”***

*We’ve clarified and added additional citations to this area.*

**Please provide a citation for this sentence *“However, simply creating a facilitated social space that respects individual communication needs may not inherently increase an individual's willingness to communicate with others.”***

*We’ve added a citation for the above sentence.*

**In the paragraph on existing uses of Minecraft and the paragraph on justification for use of Minecraft, there are some overlapping points. Please consider combining the two paragraphs. It might also be helpful to explore how Minecraft is used in therapeutic settings outside of supporting autistic individuals. For example, these articles discuss the use of Minecraft in other scenarios. Using these to support the paragraphs would be a valuable addition. The article links are provided here. Mining for Social Skills: Minecraft in Home and Therapy for Neurodiverse Youth; Gliosci & Barros Pontes E Silva ^1^ ; Jagannath, Salen & Slovàk ^2^ .**

 We appreciate the additional citations and recommendations regarding the structure and have combined the two sections, clarified the citations that describe populations other than solely autistic youth, included additional citations.

**Further in the paragraph on justification, there are points that also tie in with points in the previous paragraph on applications with neurodivergent individuals. So, I encourage the authors to re-read and consider rewriting these sections to be mutually exclusive paragraphs of the essay.**

*We’re restructured the two sections to create mutually exclusive sections.*

**The method of implementation is clear with case examples. Practical suggestions on how to facilitate the session would be helpful to add here for others to facilitate the groups if that was the intended purpose of the article.**

**Suggestions on what facilitation entails would be helpful for someone to replicate the groups as well. What is the main goal of facilitation?**

*We appreciate this feedback and have worked to clarify our language around the goal of the article as well as for facilitation. As instruction on facilitating groups is beyond the scope of this paper, we’ve refrained from adding more specific recommendations around facilitation, though we did add additional information about qualifications for facilitators.*

# Reviewer report: Reviewer response for version 1

**DOI:** 10.5256/f1000research.141749.r187083

**Published:** 2023-07-26

**Authors:** James Hutson

**Affiliations:** 1 Department of Art History and Visual Culture, Lindenwood University, Saint Charles, USA

The rationale for utilizing Minecraft as a platform for therapeutic intervention is thoroughly expounded upon within the discourse. The authors highlight the game's cultural ubiquity, popularity, and the varied gameplay modes as pivotal elements that contribute to its suitability in a therapeutic context.

 The popularity of Minecraft, especially among the target demographic, helps foster participant motivation and engagement. This facet is underscored by the argument that familiarity with the game can ease the transition into therapy and allow participants to assume the role of experts, thus facilitating their involvement in the therapy process. This facet is particularly salient when working with neurodivergent individuals, considering the existence of Minecraft servers and communities specifically designed for these individuals, such as Autcraft. 

 The description of the method for utilizing Minecraft as a therapeutic tool appears technically sound, feasible, and carefully considered for different contexts of application. The flexibility of delivery, either virtually or in-person, is an advantageous aspect, ensuring that therapy can proceed under varying circumstances.

 To supplement the in-game communication channels, the suggestion for a private shared voice and video chat for virtual meetings, or a shared table setup for in-person sessions, ensures that all participants can interact seamlessly. The consideration for such details demonstrates an understanding of the importance of effective communication for the success of the group activities.

 The structural breakdown of the sessions also appears well-conceived, with a mix of check-ins, game activities, and check-outs, enhancing the opportunity for participants to discuss their experiences and offer feedback. This approach is likely to facilitate the therapeutic process by providing routine and structure.

Is the rationale for developing the new method (or application) clearly explained?

Yes

Is the description of the method technically sound?

Yes

Are the conclusions about the method and its performance adequately supported by the findings presented in the article?

Yes

If any results are presented, are all the source data underlying the results available to ensure full reproducibility?

No source data required

Are sufficient details provided to allow replication of the method development and its use by others?

Yes

Reviewer Expertise:

AI, neurohumanities and neurodiversity

I confirm that I have read this submission and believe that I have an appropriate level of expertise to confirm that it is of an acceptable scientific standard.
